# Community-wide prevalence and intensity of soil-transmitted helminthiasis and *Schistosoma mansoni* in two districts of Sierra Leone

**DOI:** 10.1371/journal.pntd.0010410

**Published:** 2022-05-20

**Authors:** Cara Tupps, Ibrahim Kargbo-Labour, Jusufu Paye, Sanjaya Dhakal, Mary H. Hodges, Alexander H. Jones, Stacy Davlin, Mustapha Sonnie, Sallay Manah, Rubina Imtiaz, Yaobi Zhang

**Affiliations:** 1 Children Without Worms, The Task Force for Global Health, Atlanta, Georgia, United States of America; 2 Neglected Tropical Diseases Program, Ministry of Health and Sanitation, Freetown, Sierra Leone; 3 Helen Keller International, Freetown, Sierra Leone; 4 Helen Keller International, Regional Office for Africa, Dakar, Senegal; Centers for Disease Control and Prevention, UNITED STATES

## Abstract

In Sierra Leone, nationally powered school-based surveys have documented significant progress in the control of soil-transmitted helminthiasis (STH) and schistosomiasis. In order to assess the district-level prevalence and intensity of infection among key at-risk groups outside of school age children (SAC), we conducted a multi-stage, cluster-sample household survey in Bo and Kenema districts in May 2018. From both districts, we examined 1,282 pre-school age children (PSAC), 730 school age children (SAC), and 517 adults over 14 years (including 387 women of reproductive age, or WRA) for STH and *Schistosoma mansoni* infection using Kato Katz technique. In Bo, STH prevalence was 8.0% (95% Upper Confidence Limit 10.2%) in PSAC, 6.4% (95% Upper Confidence Limit 9.0%) in SAC, 14.1% (95% Upper Confidence Limit 17.4%) in all adults and 11.9% (95% Upper Confidence Limit 17.4%) in WRA. In Kenema, STH prevalence was 18.1% (95% Upper Confidence Limit 20.5%) in PSAC, 17.3% (95% Upper Confidence Limit 20.7%) in SAC, and 16.9% (95% Upper Confidence Limit 20.5%) in all adults and 16.9% (95% Upper Confidence Limit 22.6%) in WRA. Hookworm species were the most prevalent of STH in both districts overall. The overall prevalence of *S*. *mansoni* was <10% in Bo and <20% in Kenema, and was similar across age groups. No moderate or heavy intensity STH infections or heavy intensity *S*. *mansoni* infections, as per World Health Organization (WHO) classification, were detected in either district. Sanitation variables, such as toilet access and quality, were independently associated with STH and *S*. *mansoni* infection. In Kenema, STH prevalence in SAC was within the WHO-defined range for annual treatment, whereas a previous nationally-powered survey estimated it to lie within the range of treatment once per two years. By utilizing community-based sampling, we were able to assess prevalence among WRA and make recommendations based on current guidance from WHO. To continue toward elimination of STH and *S*. *mansoni* as a public health problem, resources should be mobilized to increase access to and uptake of improved sanitation at community and household levels.

## Author summary

Soil-transmitted helminthiasis (STH) and schistosomiasis affect the nutrition and development of children and pregnant women around the world and are controlled through administration of anthelminthic medication, good personal hygiene practices, and improvement of sanitation. Globally, current infection control programming provides anthelminthic medication to school age children using a school-based delivery platform. Monitoring activities for these programs also focus on school children, but global treatment recommendations also include women of reproductive age as an at-risk population. In order to assess the prevalence among this group and the community as a whole, we conducted a prevalence study of these two diseases among all age groups in Sierra Leone. We found that the STH prevalence in children warrants annual treatment in Kenema district and once every two years in Bo district, and that there is a case to consider targeting women of reproductive age for STH treatment based on the prevalence in both districts. We also found that prevalence of all species of worms was significantly lower among those who with access to toilets compared to those who did not in both districts. This highlights the need to improve sanitation at community and household levels to achieve and maintain control of these diseases.

## Introduction

Soil-transmitted helminthiasis (STH) and schistosomiasis affect over one billion people globally [1]. Schistosomiasis is primarily caused by the species *Schistosoma mansoni*, *Schistosoma haematobium*, and *Schistosoma japonicum*, while STH is primarily caused by the species *Ascaris lumbricoides*, *Trichuris trichiura*, and hookworm (*Ancylostoma duodenale* or *Necator americanus*) [2]. STH and schistosomiasis have been linked to poor nutritional status and cognitive impairment, particularly in children [2].

The World Health Organization (WHO) has targeted STH and schistosomiasis for elimination as a public health problem in pre-school age children (PSAC) and school-age children (SAC) by 2030, defined as <2% prevalence of moderate-to-heavy intensity infection (MHII) for STH, and <1% prevalence of heavy intensity infection (HII) for schistosomiasis [3]. According to WHO’s program guidelines, administration of preventive chemotherapy to SAC (5–14 years old) for the control of STH infection can be stopped when STH prevalence falls to <2% [4]. The WHO also recommends treatment of PSAC (1–4 years old) and women of reproductive age (WRA; 15–49 years old) in areas where the baseline prevalence of STH and/or schistosomiasis is at least 20% [5].

Due to the operational burden of targeting an additional population group, the decision to monitor and treat WRA varies according to programmatic resources and priorities. The norm for STH program monitoring surveys is a school-based model for logistical and cost reasons. While SAC typically carry the highest burden of STH and are accepted as a proxy for the overall burden of STH in a community, conducting surveys via schools risks missing key data points among WRA and other population groups [6,7]. This is especially true in areas where *T*. *trichiura* and hookworm species are endemic, as they are linked to an increased risk of anemia in women and young girls [8]. In contexts like Sierra Leone, where different age groups have been targeted during several disease-specific campaigns, it is important to understand the burden of STH and schistosomiasis among all age groups in order to make decisions on which populations, if any, still require treatment per WHO guidance. This information is particularly relevant for districts which have qualified for cessation of mass drug administration (MDA) for lymphatic filariasis (LF).

In Bo and Kenema, school-based STH control programs began in 2002, targeting SAC only and utilizing albendazole [9]. Ivermectin MDA for onchocerciasis control–which is also highly effective against *A*. *lumbricoides* [10]–also began that year, targeting SAC and adults in conflict displacement camps initially and in highly endemic villages by 2005 [11]. In 2006, biannual MDA targeting PSAC commenced [9]. Community-wide MDA of ivermectin plus albendazole for the elimination of LF began in 2008, reaching national scale in 2010 [9]. The schistosomiasis control program started in 2009, targeting SAC and at-risk adults [12]. All MDAs were suspended in 2014 due to the Ebola epidemic, and resumed the following year. The Sierra Leone Ministry of Health and Sanitation (MoHS), along with Helen Keller International (Helen Keller), conducted nationally powered school-based baseline surveys for STH and schistosomiasis from 2008–2011 to inform program planning [13–15]. Bo and Kenema districts were included in these surveys and were both identified as warranting treatment for both STH and schistosomiasis. Baseline prevalence for any STH species was 18.4% in Bo, and 53.3% in Kenema [14].

Results from impact surveys conducted in 2012 and 2016 showed a decrease in both STH and *S*. *mansoni* nationwide, including in Bo and Kenema districts [9,16,17]. In mid-2017, the STH control strategy for PSAC began transitioning from MDA to routine biannual treatment at health facilities. Bo and Kenema underwent this transition, and their last round of MDA targeting PSAC was in May 2017 while MDA for SAC remains in effect for both districts. Bo district qualified to cease community-wide MDA for LF in 2017, where the last round occurred in May through July of 2016 [18], and Kenema district achieved this in May 2021.

In 2018, Children Without Worms (CWW), at the request of Helen Keller and the Sierra Leone MoHS, supported population-based surveys in Bo and Kenema districts to determine the district-level prevalence and intensity of STH and *S*. *mansoni* within PSAC, SAC, and adults over 14. The purpose of conducting these surveys was to assess the added value of community-wide data powered to the unit of MDA implementation for making programmatic decisions on STH and *S*. *mansoni*, and to make treatment recommendations for populations which would be affected by phasing out community-wide MDA for LF. These districts were chosen purposively, in order to assess the community-based survey model as a surveillance tool for STH in both high prevalence and low prevalence settings, and because MDA had not yet been completed in either district. Here, we discuss these survey results and their utility for evidence-based decision making in addition to the nationally powered survey results.

## Methods

### Ethics statement

The survey protocol was reviewed by the Sierra Leone MoHS Ethics and Scientific Review Committee, which granted its approval. In the local language, formal consent was obtained before any individual participated. All participants 18 years or older provided verbal consent, and assent was obtained from parents or guardians of participants under 18 years. There was no compensation for participation. All data were anonymized and remain confidential.

### Survey design

The Integrated Community-based Survey for Program Monitoring (ICSPM) was conducted in Bo and Kenema districts, a methodology developed by CWW to estimate the prevalence and intensity of infection for STH in each age group within an evaluation unit. In Sierra Leone, the age groups were defined as follows: PSAC (1–4 years old), SAC (5–14 years old), and adults (15–49 years old). Because all adults can contribute to transmission of STH it was important to determine the overall prevalence in adults regardless of age and sex. However, since the WHO specifically includes WRA as an at-risk group for STH, results were analyzed for this sub-group as well. Diagnostic results of *S*. *mansoni* were also collected and reported.

The ICSPM is a multi-stage, cluster-sample household survey, which is powered to the district level. The survey design is self-weighting within strata and risk groups, meaning that all members of each risk group in each stratum have the same probability of being selected in the survey sample. Clusters are randomly selected from each evaluation unit (district) using probability proportional to estimated size. Census enumeration areas were chosen as the “cluster” unit, and an exhaustive list of enumeration areas within each district was obtained from the national census office. From each district, 30 clusters were selected as primary sampling units. Segmentation was also utilized to increase efficiency of household selection in the field and thus reduce costs. All selected clusters were divided into segments of approximately 75 households. In each selected cluster, one segment was then randomly selected. The full methodology has been previously described in detail [19], and is also available on the CWW website (https://childrenwithoutworms.org/icspm-reference-manual/).

Individuals from selected segments were systematically sampled to ensure a minimum sample size of 332 in each age group per district, accounting for an estimated non-response rate of 30%. The 2015 census data were used to calculate household sampling intervals for each age group in a district [20]. The sample size of 332 per age group in each district was sufficient to detect whether STH prevalence falls beneath the WHO-defined thresholds [2] down to <10% and is based on WHO sample size guidance for the Transmission Assessment Survey [21]. The ICSPM ensures that every individual has an equal probability of being enrolled and was statistically powered to assess prevalence and compare the differences between age groups at the district-level.

### Data collection

Data collection occurred in May 2018. Enrollment criteria for participants were as follows: at least 1 year old, had slept at the household the previous night, planning to stay at the household on the night of screening, ability and willingness to provide a single stool sample, and ability and willingness to provide verbal consent (for those at least 18 years of age) or assent (for those under 18 years). A household was defined as a group of people who live together and share food from a single cooking pot. Enumerators interviewed eligible respondents on their demographics and other variables. Android smart phones running Open Data Kit software were used to record data.

### Sample collection and diagnostics

Specimens were analyzed via the Kato Katz technique the same day as collection, and samples were kept in cooler boxes until they were prepared [22]. Two slides were prepared from each sample and read by different laboratory technologists. Results were verified on a random sample of slides by another technologist. An average of eggs per gram of stool (epg) was calculated from the two final readings and used to classify infections by intensity using WHO classifications [23].

### Data analysis

Analysis was carried out using STATA version 16 (StataCorp. 2019. Stata Statistical Software: Release 16. College Station, TX: StataCorp LLC.), using a design effect of 2.0. Since ICSPM produces an equal-probability sample of risk-group members within strata, all clusters in each district were selected from the same sampling frame, and analyses were kept separate for each district, no weight variables were added. One-sided 95% upper confidence limits (UCLs) were reported for measures of prevalence, including prevalence of MHII and HII, as the survey is designed to determine the lowest threshold under which the true prevalence lies [19]. Two-sided 95% confidence intervals (CIs) were calculated for all other estimates.

Each variable was analyzed for a statistically significant association with STH infection using Pearson’s chi-squared test with the Rao and Scott correction. For all statistical computations, a p-value of <0.05 was considered significant. Crude prevalence ratios for STH by age group and all other variables were calculated using generalized linear models with binomial distribution and a log-link function. Spatial correlation of positive results for STH was tested using Moran’s I and cluster distribution was plotted using ArcGIS version 10 (ESRI, Redlands, California, United States).

## Results

### Age and sex of respondents

We enrolled 3,685 individuals (52% females, 48% males, <1% unreported). Overall, 2,779 (75%) of those enrolled provided stool samples, and we could link 2,692 (54% females, 46% males, <1% unreported) diagnostic results to individual questionnaire responses. We were unable to link the remaining 3% of laboratory results to any enrolled individual, so we excluded these from analysis. This was likely due to mislabeled samples, data entry errors, or both.

From Bo, 1,193 samples were analyzed and 1,499 were analyzed from Kenema. Participant age ranged from 1–90 years. In both districts, female participation was higher than males across all age groups (Table 1). In Bo, there was no significant difference in the mean age of males compared to females (p>0.20).

**Table 1:** Age and sex breakdown of respondents by district.

	Bo District			Kenema District		
	Female	Male	Overall	Female	Male	Overall
	n (%)	n (%)	n (%)	n (%)	n (%)	n (%)
**Age group**						
PSAC (1–4 years)	249 (50.4)	245 (49.6)	503^a^ (42.2)	413 (53.0)	366 (47.0)	779 (52.0)
SAC (5–14 years)	189 (55.3)	153 (44.7)	342 (28.7)	204 (52.6)	184 (47.4)	388 (25.9)
Adults (>14 years)	185 (53.2)	163 (46.8)	348 (29.2)	202 (60.8)	130 (39.2)	332 (22.2)

PSAC: pre-school age children; SAC: school-age children; (a) Sex was not reported for nine PSAC.

### Prevalence and intensity of STH and *S*. *mansoni* infections

**Fig 1:**
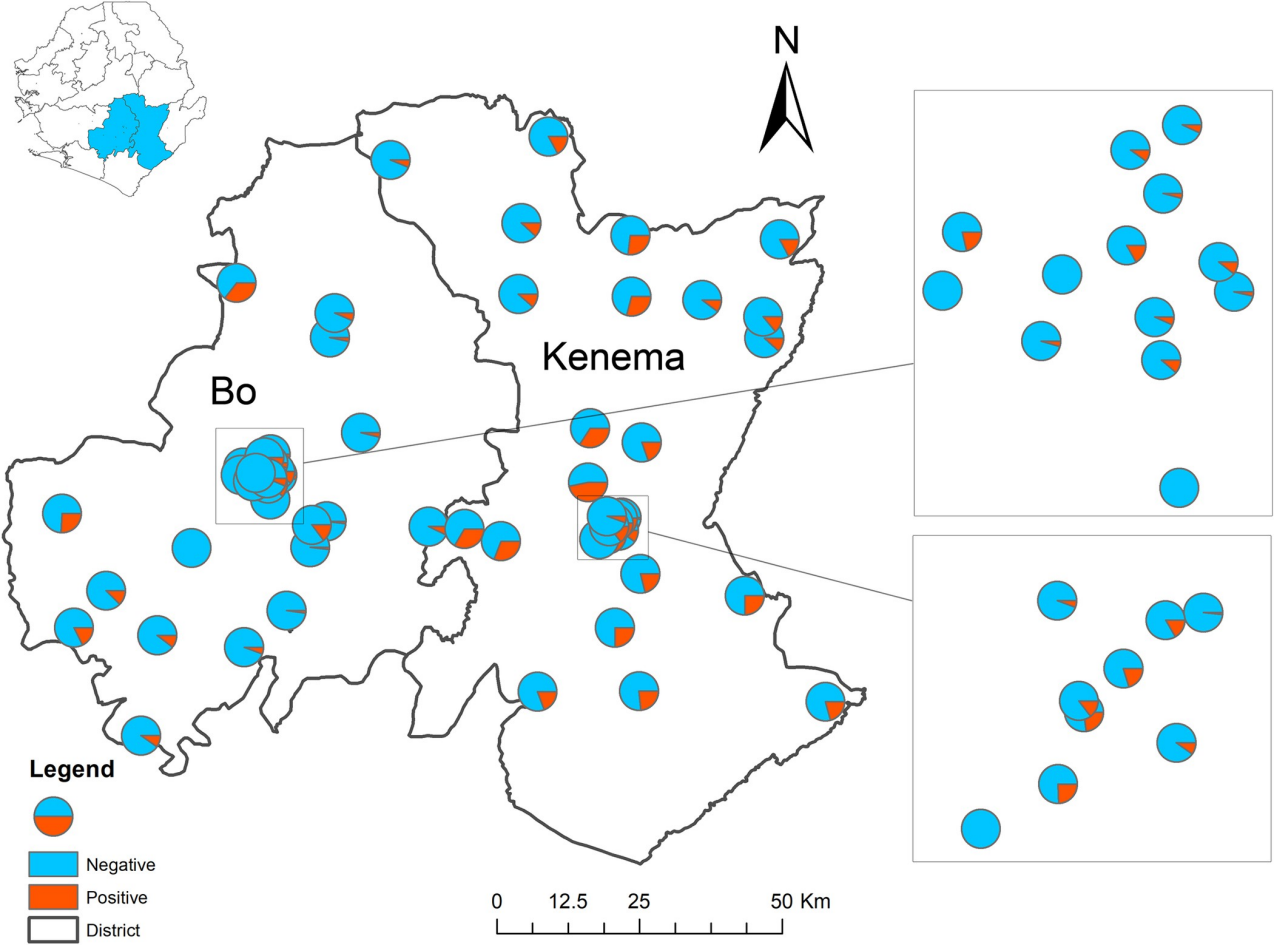
Map of survey clusters and the distribution of diagnostic results for soil transmitted helminthiasis. The base layer for this map was obtained from the GADM database (www.gadm.org), version 2.8: https://www.gadm.org/download_country_v2.html.

**Fig 2:**
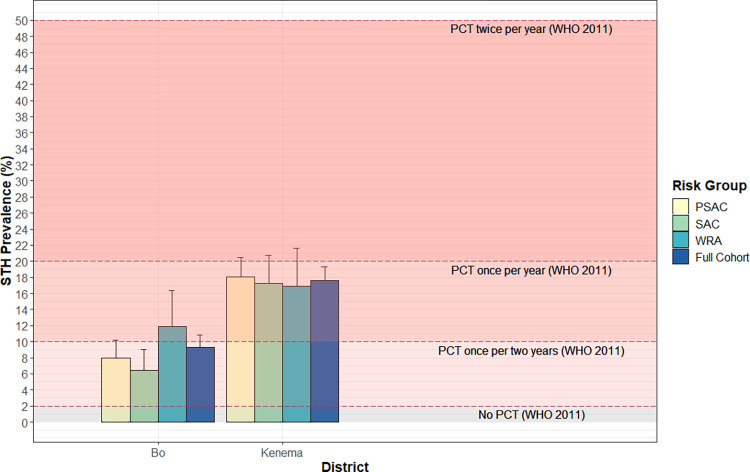
Soil transmitted helminthiasis prevalence by World Health Organization target risk group and thresholds for post-reassessment MDA frequency. STH: soil transmitted helminthiasis; PSAC: pre-school age children; SAC: school-age children; WRA: Women of Reproductive Age; Prevalence thresholds taken from *Helminth control in school-age children*: *A guide for managers of control programmes*. *2nd ed*. *Geneva*: *World Health Organization; 2011*, *p*.*74*.

The proportion of positive STH results within each survey cluster is shown in Fig 1. The overall STH prevalence was 9.3% (95% UCL 10.8%) in Bo and 17.6% (95% UCL 19.3%) in Kenema (Table 2). *A*. *lumbricoides* and hookworm were the most prevalent STH species (Table 2). In both districts, all STH infections were light as per WHO classification [23]. STH prevalence was similar by sex in both districts (p>0.27 in Bo and p>0.46 in Kenema). Age was associated with increased prevalence in Bo district, with significantly higher prevalence among adults compared to SAC (Table 3). No trend with age was observed in Kenema district (S1 Table). STH prevalence among WRA was 11.9% (17.4% UCL) in Bo and 16.8% (22.6% UCL) in Kenema (Fig 2), and was similar to that of all adults in both districts.

**Table 2:** Prevalence of infection for all species by age group and district.

		Bo District ^a^		Kenema District		
		Prevalence	Mean Epg		Prevalence	Mean Epg
		(95% UCL)	(95% CI)		(95% UCL)	(95% CI)
**PSAC**	**n = 503**			**n = 779**		
Any STH		8.0 (10.2)			18.1 (20.5)	
*A*. *lumbricoides*		4.6 (6.4)	30.1 (13.6–46.7)		8.4 (10.1)	49.3 (40.5–58.1)
*T*. *trichiura*		1.2 (2.3)	84.0 (0–213.3)		0.9 (1.7)	36.9 (29.9–43.8)
Hookworm spp		3.6 (5.2)	90.9 (14.2–167.5)		10.5 (12.5)	59.4 (48.5–70.3)
*S*. *mansoni*		1.4 (3.4)	268.8 (0–761.6)		12.7 (14.8)	75.9 (63.4–88.5)
**SAC**	**n = 342**			**n = 388**		
Any STH		6.4 (9.0)			17.3 (20.7)	
*A*. *lumbricoides*		2.9 (4.9)	61.3 (8.8–113.8)		5.9 (8.2)	40.7 (32.3–49.1)
*T*. *trichiura*		0.6 (1.9)	192.0 (0–397.9)		1.0 (2.3)	36.0 (26.2–45.8)
Hookworm spp		3.8 (5.9)	136.4 (30.8–241.9)		10.8 (13.7)	58.6 (44.6–72.6)
*S*. *mansoni*		1.8 (3.4)	120.0 (0–307.8)		13.9 (17.1)	81.3 (63.1–99.6)
**Adults**	**n = 348**			**n = 332**		
Any STH		14.1 (17.4)			16.9 (20.5)	
*A*. *lumbricoides*		4.9 (7.2)	62.0 (31.3–92.7)		7.8 (10.6)	37.4 (29.9–44.9)
*T*. *trichiura*		2.0 (3.7)	51.4 (22.9–79.9)		1.0 (2.3)	33.0 (20.6–45.4)
Hookworm spp		8.9 (11.8)	100.2 (34.0–166.5)		9.0 (12.0)	45.8 (33.4–58.2)
*S*. *mansoni*		1.1 (2.6)	48.0 (17.1–78.9)		16.0 (19.6)	71.0 (51.7–90.4)
**Overall**	**n = 1,193**			**n = 1,499**		
Any STH		9.3 (10.8)			17.6 (19.3)	
*A*. *lumbricoides*		4.2 (5.3)	45.7 (29.2–62.2)		7.6 (8.8)	44.8 (39.2–50.5)
*T*. *trichiura*		1.3 (1.9)	83.2 (22.9–143.5)		0.9 (1.4)	44.0 (26.7–61.3)
Hookworm spp		5.2 (6.4)	104.3 (58.1–150.5)		10.3 (11.6)	56.6 (49.2–63.9)
*S*. *mansoni*		1.4 (2.1)^a^	168 (0–393.2)^a^		13.8 (15.3)	76.2 (67.0–85.3)

PSAC: pre-school age children; STH: soil transmitted helminths; SAC: school-age children; UCL: upper confidence limit. Epg: eggs per gram of stool; (a) There were few positive cases of *S*. *mansoni* in Bo district (n = 5 for PSAC; n = 3 for SAC; n = 3 for adults) and results should be interpreted with caution; NB: The lower threshold of sensitivity for Kato Katz is 10%, thus all results <10% should be interpreted with caution.

**Table 3:** Distribution of soil transmitted helminthiasis cases (any species) and crude prevalence ratios for variables independently associated with infection, both districts.

	Bo District				Kenema District			
	STH pos. (%)	STH neg. (%)	PR	95% CI	STH pos. (%)	STH neg. (%)	PR	95% CI
**Age Group**								
SAC (5–14 years)	22 (6.4)	320 (93.6)	1.00		67 (17.3)	321 (82.7)	1.00	
PSAC (1–4 years)	40 (8.0)	463 (92.0)	1.24	0.75–2.04	141 (18.1)	638 (81.8)	1.05	0.81–1.37
Adults (>14 years)	49 (8.0)	299 (92.0)	2.19**	1.35–3.54	56 (16.9)	276 (83.1)	0.98	0.71–1.35
**Other Variables**								
Student	41 (8.0)	469 (92.0)	0.86	0.59–1.25	66 (22.8)	224 (77.2)	1.39*	1.09–1.78
Farmer	10 (13.2)	66 (86.8)	1.55	0.84–2.86	21 (28.4)	53 (71.6)	1.66*	1.14–2.43
Rural location	74 (10.6)	614 (89.4)	1.47*	1.01–2.14	187 (20.7)	717 (79.3)	1.60***	1.25–2.04
Disposal of child stools in bush	53 (16.4)	270 (83.6)	2.46***	1.73–3.49	86 (25.6)	250 (74.4)	1.67***	1.33–2.10
No toilet facility at home	54 (17.5)	254 (82.5)	2.72***	1.92–3.86	75 (25.4)	220 (74.6)	1.62**	1.28–2.05
Improved toilet at home	40 (6.0)	624 (94.0)	0.45***	0.31–0.65	135 (14.4)	800 (85.6)	0.63**	0.51–0.78
Improved toilet outside home	61 (8.1)	691 (91.9)	0.72	0.50–1.02	131 (14.4)	780 (85.6)	0.64***	0.51–0.79

PR: crude prevalence ratio; CI: confidence interval; PSAC: pre-school age children; SAC: school-age children

*p<0.05

**p<0.01

***p<0.001.

No clustering effect for STH prevalence was shown within either Bo (Moran’s I = -0.049309, Z-score = -0.17407, p-value = 0.86) or Kenema district (Moran’s I = 0.013128, Z-score = 0.284335, p-value = 0.776). Analysis of both districts combined did show a significant clustering effect between districts (Moran’s I = 0.192695, Z-score = 2.180256, p-value = 0.029238).

For *S*. *mansoni*, the overall prevalence was 1.4% (95% UCL 2.1%) in Bo and 13.8% (95% UCL 15.3%) in Kenema (Table 2). No HII cases were detected for *S*. *mansoni*. The prevalence was similar across age groups (p = 0.98 in Bo and p = 0.35 in Kenema) and sex (p = 0.86 in Bo and p = 0.08 in Kenema).

### Variables associated with STH infection

The full results for unadjusted STH prevalence ratios by the variables included in this study are available in S1 Table. Variables found to be associated with higher STH prevalence in either district were profession, sanitation, and rural location. Prevalence among students and farmers was higher relative to all other professions in Kenema (Table 3), but no significant trends by profession were found in Bo (S1 Table).

STH prevalence was higher among respondents without access to any toilet facilities at home in both districts (Table 3). There was lower STH prevalence among those with access to improved toilet facilities, as defined by UNICEF and the WHO [24]. In Bo and Kenema, 308 (26%) and 295 (20%) respondents lacked any toilet facilities at home, respectively. Adults with children under five in the household who reported disposal of the children’s stools in the bush had a higher prevalence of STH infection relative to those who disposed of them in the toilet or buried them (Table 3).

STH prevalence was significantly higher among those living in rural areas (located outside of town boundaries) compared to the non-rural areas in each district (Table 3). In Bo, 407 (59%) rural respondents and 478 (95%) of non-rural respondents had a toilet at home. In Kenema, 609 (67%) rural respondents and 595 (100%) non-rural respondents had a toilet at home. For improved facilities, 240 (35%) of rural respondents in Bo and 343 (38%) in Kenema reportedly had access compared to 424 (84%) of non-rural respondents in Bo and 592 (>99%) in Kenema.

### Variables associated with *S*. *mansoni* infection

The full results for unadjusted *S*. *mansoni* prevalence ratios by the variables included in this study are available in S2 Table. This analysis was not conducted for *S*. *mansoni* in Bo due to the low number of cases (n = 11). Variables found to be associated with higher *S*. *mansoni* prevalence in Kenema district were profession and sanitation. Prevalence of *S*. *mansoni* was higher among those who indicated their primary profession to be farming or house work compared to all other professions (Table 4). Prevalence was significantly higher among those without access to any toilet facility at home compared to those with some facility, and was lower among those with an improved toilet facility at home compared to those with unimproved facilities (Table 4). Higher prevalence was also observed among those who disposed of the children’s stools in the bush compared to those who disposed of them in the toilet or buried them (Table 4).

**Table 4:** Distribution of *S*. *mansoni* cases and crude prevalence ratios for variables independently associated with infection, Kenema district.

	Kenema District			
	*S*. *mansoni* pos. (%)	*S*. *mansoni* neg. (%)	PR	95% CI
**Age Group**				
SAC (5–14 years)	54 (13.9)	334 (86.1)	1.00	
PSAC (1–4 years)	101 (13.0)	678 (87.0)	0.92	0.65–1.31
Adults (>14 years)	54 (16.3)	278 (83.7)	1.20	0.80–1.81
**Other Variables**				
Farmer	22 (29.7)	52 (70.3)	2.27***	1.56–3.30
House work	23 (24.2)	72 (75.8)	1.83**	1.25–2.67
Disposal of child stools in bush	59 (17.6)	277 (82.4)	1.36*	1.03–1.79
No toilet facility at home	55 (18.6)	240 (81.4)	1.46**	1.10–1.93
Improved toilet at home	117 (12.5)	818 (87.5)	0.76*	0.60–0.99

PR: crude prevalence ratio; CI: confidence interval; PSAC: pre-school age children; SAC: school-age children

*p<0.05

**p<0.01

***p<0.001.

### MDA coverage and platform

In Bo district, self-reported MDA coverage within the past 12 months across the three age groups (PSAC, SAC and adults) was 47.6%, 54.7%, and 59.5% in Bo and 52.4%, 60.3% and 61.4% in Kenema, respectively. Self-purchase of treatment was reported among 9.0% of respondents who reported receipt of MDA in Bo and 21.3% in Kenema.

## Discussion

STH prevalence results show that the recommended MDA frequency according to the 2011 WHO reassessment guidelines [2] would be once every two years in Bo and once every year in Kenema overall, and among PSAC and SAC populations specifically (Fig 2). In Bo district, our results for SAC were similar to those of the 2016 national survey. In Kenema, one result which equated to a different recommended treatment frequency was that we estimated STH prevalence among SAC to be 17.3% in 2018, while the 2016 survey estimated prevalence had fallen below the 10% threshold for annual treatment (6.6%; 95% CI 4.3–10.0%) [9]. Our results support the national program’s declaration that STH has been eliminated as a public health problem in both districts, as only light intensity infections were detected, and affirm the national program’s success in targeting this disease.

Self-reported MDA coverage was lower than national MDA coverage data reported for 2017–2018, which was over 88% for SAC in both districts [25]. Memory limitations may explain this difference, as over six months had elapsed since the last MDA. In Kenema, which shares a border with Liberia, dynamic population denominators may also partially account for this difference, as has been indicated by LF monitoring surveys [18].

According WHO, preventive chemotherapy for WRA is necessary where baseline prevalence is ≥20% [5]. As the survey took place after many rounds of MDA, our results cannot be used as a baseline for this population and as such the decision for treatment of WRA should follow WHO’s 2011 reassessment guideline [2]. In both districts, our results suggest that annual MDA is warranted among WRA if following this guideline (Fig 2). However, WHO’s 2017 guideline states that the recommendations are based on the assumption that only light intensity infection is expected where baseline prevalence is below 20% [5], and all STH infections were light in both districts. Although the guidelines are difficult to interpret for this group, hookworm was the most prevalent species among adults in both districts with no difference by sex. For this reason we still recommend targeting WRA for STH treatment in both districts in order to continue to control morbidity among WRA and reach WHO’s global 2030 targets for neglected tropical diseases [4].

The potential impact of improved household-level sanitation was shown in both districts by the association between STH and *S*. *mansoni* prevalence, access to toilet facilities, and the disposal method of young children’s stools. Safe stool disposal facilities at the household level, new toilets, improvements to existing toilets, and promoting the behavioral changes necessary to consistently use and maintain any new facilities could reduce the prevalence of STH and *S*. *mansoni* in these districts.

The heterogeneity of prevalence and significant clustering between the districts suggests that district-level surveys may help identify factors and/or population groups driving infection. In both districts, for example, concentrating on sanitation interventions in rural locations would have a significant and sustainable impact on STH control. District level data such as these can inform programmatic decision making more precisely as programs look to identify and address areas of persistently high infection, and as prevalence approaches the threshold to stop MDA.

### Limitations

As the survey methodology was designed primarily to detect community-wide STH prevalence, we did not use purposive or spatially-driven sampling which is optimal for the detection of schistosomiasis infection. We excluded certain information from the analysis, such as household water sources and flooring materials, as we were not confident in linking these variables to the diagnostic data.

We did not conduct stratified or multivariate analysis of sanitation or other variables, as our primary objective was to assess the added value of community-wide prevalence data at the sub-national level for programmatic decision making. The variables included in the survey are known risk factors for STH and schistosomiasis, and their inclusion serves to help program managers assess what specific factors may be contributing to the transmission of these diseases. A robust analysis of this data would be ideal in settings where transmission remains high despite continued treatment at high coverage levels.

The survey was set up to obtain a statistically representative sample of all adults within an evaluation unit and not WRA specifically. However, the prevalence for all adults was similar to that for WRA and there was no significant difference in prevalence in adults by sex. Thus, we have included the prevalence results for WRA as they provide a previously missing picture of STH prevalence among a key at-risk group. Finally, due to the diagnostic sensitivity limitations of Kato Katz the most appropriate interpretation of prevalence measures below 10% is that they are confirmed to fall below that threshold by the UCL [26].

### Conclusions

We were able to assess STH prevalence and intensity among different at-risk groups, including WRA, and generate evidence-based MDA recommendations for the district level in all age groups. In Kenema district, these results did result in a different recommended treatment frequency in SAC than the national survey results.

Based on these results, we recommend that STH programs be established and/or scaled up to target WRA in both districts. As community-wide MDA for LF in Kenema district phases out, we recommend using the next coverage survey for STH and schistosomiasis to identify areas at the sub-district level where low coverage or non-compliance in hard-to-reach populations may be driving transmission. Finally, initiatives to improve sanitation access, quality, and use should be included in efforts to continue controlling STH and *S*. *mansoni* infection. It will be critical for these initiatives to integrate robust social engagement strategies to promote uptake and utility.

Although community-based surveys are not the norm in STH surveillance, we have shown that they provide important insights into transmission dynamics which can be used to geographically target and optimize delivery of MDA, sanitation, and other STH control interventions. The ICSPM survey can be used as a practical data collection tool for such program strategies in the future. As community-based surveys are more complex than school-based surveys, they are best utilized where national programs are considering a geographical and/or risk-group targeted approach to eliminate STH as a public health problem, and where adults are suspected to be a significant driver of infection. We believe the need for district-level, community-based data such as these will only become more essential to evidence-based decision making as national programs approach the “end game” for STH control and elimination.

## Supporting information

S1 Table Distribution of soil-transmitted helminth cases across age, sex, and other variables of interest (n = 1,193 in Bo and n = 1,499 in Kenema). Each variable was analyzed for a statistically significant association with STH infection using Pearson’s chi-squared test. Crude prevalence ratios were calculated for all variables except for those with <10 observations. Prevalence ratios with * denotes a p<0.05, ** denotes a p<0.01, and *** denotes a p<0.001. STH: soil-transmitted helminth; PR: crude prevalence ratio; CI: confidence interval; SAC: school-age children; PSAC: preschool-age children; ^a^ Results should be interpreted with caution for this variable as the observations were <10 per cell; ^b^ Prevalence ratios were not calculated for variables with observations <10.
(DOCX)

S2 Table Distribution of *Schistosomiasis mansoni* cases across age, sex, and other variable of interest (n = 1,499 in Kenema). Each variable was analyzed for a statistically significant association with *S*. *mansoni* infection using Pearson’s chi-squared test. Crude prevalence ratios were calculated for all variables except for those with <10 observations. Prevalence ratios with * denotes a p<0.05, ** denotes a p<0.01, and *** denotes a p<0.001. PR: crude prevalence ratio; CI: confidence interval; SAC: school-age children; PSAC: preschool-age children; ^a^ Results should be interpreted with caution for this variable as the observations were <10 per cell; ^b^ Prevalence ratios were not calculated for variables with observations <10.
(DOCX)
