# Supplementary material for: Community-wide prevalence and intensity of soil-transmitted helminthiasis and Schistosoma mansoni in two districts of Sierra Leone
Source: PLoS Negl Trop Dis. 2022 May 20;16(5):e0010410. doi: 10.1371/journal.pntd.0010410 (PMC9162327; doi:10.1371/journal.pntd.0010410)
Supplement: S1 Table — Each variable was analyzed for a statistically significant association with STH infection using Pearson’s chi-squared test. Crude prevalence ratios were calculated for all variables except for those with <10 observations. Prevalence ratios with * denotes a p<0.05, ** denotes a p<0.01, and *** denotes a p<0.001. STH: soil-transmitted helminth; PR: crude prevalence ratio; CI: confidence interval; SAC: school-age children; PSAC: preschool-age children; a Results should be interpreted with caution for this variable as the observations were <10 per cell; b Prevalence ratios were not calculated for variables with observations <10. (DOCX) [file pntd.0010410.s001.docx]

**S1 Table**. Distribution of soil-transmitted helminth cases across age, sex, and other variables of interest (n=1,193 in Bo and n=1,499 in Kenema). Each variable was analyzed for a statistically significant association with STH infection using Pearson’s chi-squared test. Crude prevalence ratios were calculated for all variables except for those with <10 observations. Prevalence ratios with * denotes a p<0.05, ** denotes a p<0.01, and *** denotes a p<0.001.

|  | **Bo District** | | | |  | **Kenema District** | | | |
| --- | --- | --- | --- | --- | --- | --- | --- | --- | --- |
|  | STH pos. (%) | STH neg. (%) | PR | 95% CI |  | STH pos. (%) | STH neg. (%) | PR | 95% CI |
| **Sex** |  |  |  |  |  |  |  |  |  |
| Male | 56 (9.8) | 514 (90.2) | 1.00 |  |  | 144 (17.6) | 675 (82.4) | 1.00 |  |
| Female | 55 (8.8) | 568 (91.2) | 0.90 | 0.64-1.28 |  | 120 (17.6) | 560 (82.4) | 1.00 | 0.81-1.25 |
| **Age Group** |  |  |  |  |  |  |  |  |  |
| SAC (5-14 years) | 22 (6.4) | 320 (93.6) | 1.00 |  |  | 67 (17.3) | 321 (82.7) | 1.00 |  |
| PSAC (1-4 years) | 40 (8.0) | 463 (92.0) | 1.24 | 0.75-2.04 |  | 141 (18.1) | 638 (81.8) | 1.05 | 0.81-1.37 |
| Adults (>14 years) | 49 (8.0) | 299 (92.0) | 2.19** | 1.35-3.54 |  | 56 (16.9) | 276 (83.1) | 0.98 | 0.71-1.35 |
| **Primary Profession** |  |  |  |  |  |  |  |  |  |
| Non-student | 61 (9.4) | 591 (90.6) | 1.00 |  |  | 198 (16.4) | 1011 (83.6) | 1.00 |  |
| Student | 41(8.0) | 469 (92.0) | 0.86 | 0.59-1.25 |  | 66 (22.8) | 224 (77.2) | 1.39* | 1.09-1.78 |
|  |  |  |  |  |  |  |  |  |  |
| Non-farmer | 92 (8.5) | 994 (91.5) | 1.00 |  |  | 21 (8.0) | 243 (92.0) | 1.00 |  |
| Farmer | 10 (13.2) | 66 (86.8) | 1.55 | 0.84-2.86 |  | 21 (28.4) | 53 (71.6) | 1.66* | 1.14-2.43 |
|  |  |  |  |  |  |  |  |  |  |
| Non-house work | 104 (9.4) | 1001 (90.6) | 1.00 |  |  | 245 (17.5) | 1159 (82.5) | 1.00 |  |
| House work^a^ | 7 (8.0) | 81 (90.2) | 0.85 | 0.41-1.76 |  | 19 (20.0) | 76 (80.0) | 1.15 | 0.75-1.74 |
|  |  |  |  |  |  |  |  |  |  |
| Non-business person | 104 (9.2) | 1030 (90.8) | 1.00 |  |  | 257 (17.6) | 1201 (82.4) | 1.00 |  |
| Business person (formal sector)^a^ | 7 (11.9) | 52 (88.1) | 1.29 | 0.63-2.66 |  | 7 (17.1) | 34 (82.9) | 0.97 | 0.49-1.92 |
|  |  |  |  |  |  |  |  |  |  |
| Non-laborer^b^ | 108 (9.1) | 1077 (90.8) |  |  |  | 263 (17.6) | 1226 (82.4) | 1.00 |  |
| Laborer (informal sector)^b,a^ | 3 (37.5) | 5 (62.5) |  |  |  | 1 (10.0) | 9 (90.0) | 0.57 | 0.88-3.65 |
|  |  |  |  |  |  |  |  |  |  |
| Non-fisher person^b^ | 111 (9.3) | 1082 (90.7) |  |  |  | 263 (17.6) | 1234 (82.4) | 1.00 |  |
| Fisher person^b^ | - | - |  |  |  | 1 (50.0) | 1 (50.0) |  |  |
| **Location** |  |  |  |  |  |  |  |  |  |
| Non-rural location | 37 (7.3) | 468 (92.7) | 1.00 |  |  | 77 (12.9) | 518 (87.1) | 1.00 |  |
| Rural location | 74 (10.6) | 614 (89.4) | 1.47* | 1.01-2.14 |  | 187 (20.7) | 717 (79.3) | 1.60*** | 1.25-2.04 |
|  |  |  |  |  |  |  |  |  |  |
| **Footwear** |  |  |  |  |  |  |  |  |  |
| Always wears shoes outside home | 43 (9.1) | 432 (90.9) | 1.00 |  |  | 192 (18.9) | 824 (81.1) | 1.00 |  |
| Sometimes or never wears shoes outside home | 68 (9.5) | 650 (90.5) | 1.05 | 0.73-1.50 |  | 72 (14.9) | 411 (85.1) | 0.79 | 0.62-1.01 |
|  |  |  |  |  |  |  |  |  |  |
| Wears closed shoes^a^ | 2 (10.0) | 18 (90.0) | 1.00 |  |  | 16 (12.1) | 116 (87.9) | 1.00 |  |
| Wears open shoes | 109 (9.4) | 1054 (90.6) | 0.94 | 0.25-3.53 |  | 248 (18.1) | 1119 (81.9) | 1.50 | 0.93-2.40 |
|  |  |  |  |  |  |  |  |  |  |
| **Sanitation** |  |  |  |  |  |  |  |  |  |
| Disposal of child stools in toilet or burial | 58 (6.7) | 812 (93.3) | 1.00 |  |  | 178 (15.3) | 985 (84.7) | 1.00 |  |
| Disposal of child stools in bush | 53 (16.4) | 270 (83.6) | 2.46*** | 1.73-3.49 |  | 86 (25.6) | 250 (74.4) | 1.67*** | 1.33-2.10 |
|  |  |  |  |  |  |  |  |  |  |
| Any toilet facility at home | 57 (6.4) | 828 (93.6) | 1.00 |  |  | 189 (15.7) | 1015 (84.3) | 1.00 |  |
| No toilet facility at home | 54 (17.5) | 254 (82.5) | 2.72*** | 1.92-3.86 |  | 75 (25.4) | 220 (74.6) | 1.62*** | 1.28-2.05 |
|  |  |  |  |  |  |  |  |  |  |
| Unimproved toilet at home | 71 (13.4) | 458 (86.6) | 1.00 |  |  | 129 (22.9) | 435 (77.1) | 1.00 |  |
| Improved toilet at home | 40 (6.0) | 624 (94.0) | 0.45*** | 0.31-0.65 |  | 135 (14.4) | 800 (85.6) | 0.63*** | 0.51-0.78 |
|  |  |  |  |  |  |  |  |  |  |
| Unimproved toilet outside home | 50 (11.3) | 391 (88.7) | 1.00 |  |  | 133 (22.6) | 455 (77.4) | 1.00 |  |
| Improved toilet outside home | 61 (8.1) | 691 (91.9) | 0.72 | 0.50-1.02 |  | 131 (14.4) | 780 (85.6) | 0.64*** | 0.51-0.79 |

STH: soil-transmitted helminth; PR: crude prevalence ratio; CI: confidence interval; SAC: school-age children; PSAC: preschool-age children;
^a^ Results should be interpreted with caution for this variable as the observations were <10 per cell.
^b^ Prevalence ratios were not calculated for variables with observations <10.
