# Supplementary material for: Community-wide prevalence and intensity of soil-transmitted helminthiasis and Schistosoma mansoni in two districts of Sierra Leone
Source: PLoS Negl Trop Dis. 2022 May 20;16(5):e0010410. doi: 10.1371/journal.pntd.0010410 (PMC9162327; doi:10.1371/journal.pntd.0010410)
Supplement: S2 Table — Each variable was analyzed for a statistically significant association with S. mansoni infection using Pearson’s chi-squared test. Crude prevalence ratios were calculated for all variables except for those with <10 observations. Prevalence ratios with * denotes a p<0.05, ** denotes a p<0.01, and *** denotes a p<0.001. PR: crude prevalence ratio; CI: confidence interval; SAC: school-age children; PSAC: preschool-age children; a Results should be interpreted with caution for this variable as the observations were <10 per cell; b Prevalence ratios were not calculated for variables with observations <10. (DOCX) [file pntd.0010410.s002.docx]

**S2 Table**. Distribution of *Schistosomiasis mansoni* cases across age, sex, and other variables of interest (n=1,499 in Kenema). Each variable was analyzed for a statistically significant association with *S. mansoni* infection using Pearson’s chi-squared test. Crude prevalence ratios were calculated for all risk factors except for those with <10 observations. Prevalence ratios with * denotes a p<0.05, ** denotes a p<0.01, and *** denotes a p<0.001.

|  | **Kenema District** | | | |  |
| --- | --- | --- | --- | --- | --- |
|  | *S. mansoni* pos. (%) | *S. mansoni* neg. (%) | PR | 95% CI | |
| **Sex** |  |  |  |  | |
| Male | 83 (12.2) | 597 (87.8) | 1.00 |  | |
| Female | 126 (15.4) | 693 (84.6) | 1.26 | 0.97-1.63 | |
| **Age Group** |  |  |  |  | |
| SAC (5-14 years) | 54 (13.9) | 334 (86.1) | 1.00 |  | |
| PSAC (1-4 years) | 101 (13.0) | 678 (87.0) | 0.92 | 0.65-1.31 | |
| Adults (>14 years) | 54 (16.3) | 278 (83.7) | 1.20 | 0.80-1.81 | |
| **Primary Profession** |  |  |  |  | |
| Non-student | 161 (13.3) | 1048 (86.7) | 1.00 |  | |
| Student | 48 (16.6) | 242 (83.4) | 1.24 | 0.92-1.67 | |
|  |  |  |  |  | |
| Non-farmer | 187 (13.1) | 1238 (86.9) | 1.00 |  | |
| Farmer | 22 (29.7) | 52 (70.3) | 2.27*** | 1.56-3.30 | |
|  |  |  |  |  | |
| Non-house work | 186 (13.2) | 1218 (86.8) | 1.00 |  | |
| House work | 23 (24.2) | 72 (75.8) | 1.83** | 1.25-2.67 | |
|  |  |  |  |  | |
| Non-business person | 202 (13.9) | 1256 (86.1) | 1.00 |  | |
| Business person (formal sector)^a^ | 7 (17.1) | 34 (82.9) | 1.23 | 0.62-2.45 | |
|  |  |  |  |  | |
| Non-laborer | 209 (14.0) | 1280 (86.0) |  |  | |
| Laborer (informal sector)^b^ | 0 (0.0) | 10 (100.0) |  |  | |
|  |  |  |  |  | |
| Non-fisher person | 209 (14.0) | 1288 (86.0) |  |  | |
| Fisher person^b^ | 0 (0.0) | 2 (100.0) |  |  | |
| **Location** |  |  |  |  | |
| Non-rural location | 82 (13.8) | 513 (86.2) | 1.00 |  | |
| Rural location | 127 (14.0) | 777 (86.0) | 1.02 | 0.79-1.32 | |
|  |  |  |  |  | |
| **Footwear** |  |  |  |  | |
| Always wears shoes outside home | 66 (13.7) | 417 (86.3) | 1.00 |  | |
| Sometimes or never wears shoes outside home | 143 (14.1) | 873 (85.9) | 1.03 | 0.79-1.35 | |
|  |  |  |  |  | |
| Wears closed shoes | 17 (12.9) | 115 (87.1) | 1.00 |  | |
| Wears open shoes | 192 (14.0) | 1175 (86.0) | 1.09 | 0.69-1.73 | |
|  |  |  |  |  | |
| **Sanitation** |  |  |  |  | |
| Disposal of child stools in toilet or burial | 150 (12.9) | 1013 (87.1) | 1.00 |  | |
| Disposal of child stools in bush | 59 (17.6) | 277 (82.4) | 1.36* | 1.03-1.79 | |
|  |  |  |  |  | |
| Any toilet facility at home | 154 (12.8) | 1050 (87.2) | 1.00 |  | |
| No toilet facility at home | 55 (18.6) | 240 (81.4) | 1.46** | 1.10-1.93 | |
|  |  |  |  |  | |
| Unimproved toilet at home | 92 (16.3) | 472 (83.7) | 1.00 |  | |
| Improved toilet at home | 117 (12.5) | 818 (87.5) | 0.76* | 0.60-0.99 | |
|  |  |  |  |  | |
| Unimproved toilet outside home | 92 (15.6) | 496 (84.4) | 1.00 |  | |
| Improved toilet outside home | 117 (12.8) | 794 (87.2) | 0.82 | 0.64-1.06 | |

PR: crude prevalence ratio; CI: confidence interval; SAC: school-age children; PSAC: preschool-age children;
^a^ Results should be interpreted with caution for this variable as the observations were <10 per cell.
^b^ Prevalence ratios were not calculated for variables with observations <10.
